# Supplementary material for: Epithelial invagination by a vertical telescoping cell movement in mammalian salivary glands and teeth
Source: Nat Commun. 2020 May 12;11:2366. doi: 10.1038/s41467-020-16247-z (PMC7217937; doi:10.1038/s41467-020-16247-z)
Supplement: Supplementary file 5 — Supplementary Information [file 41467_2020_16247_MOESM5_ESM.pdf]

Supplementary Information

This file includes:
Supplementary Figs.1 to 4
Supplementary Table 1

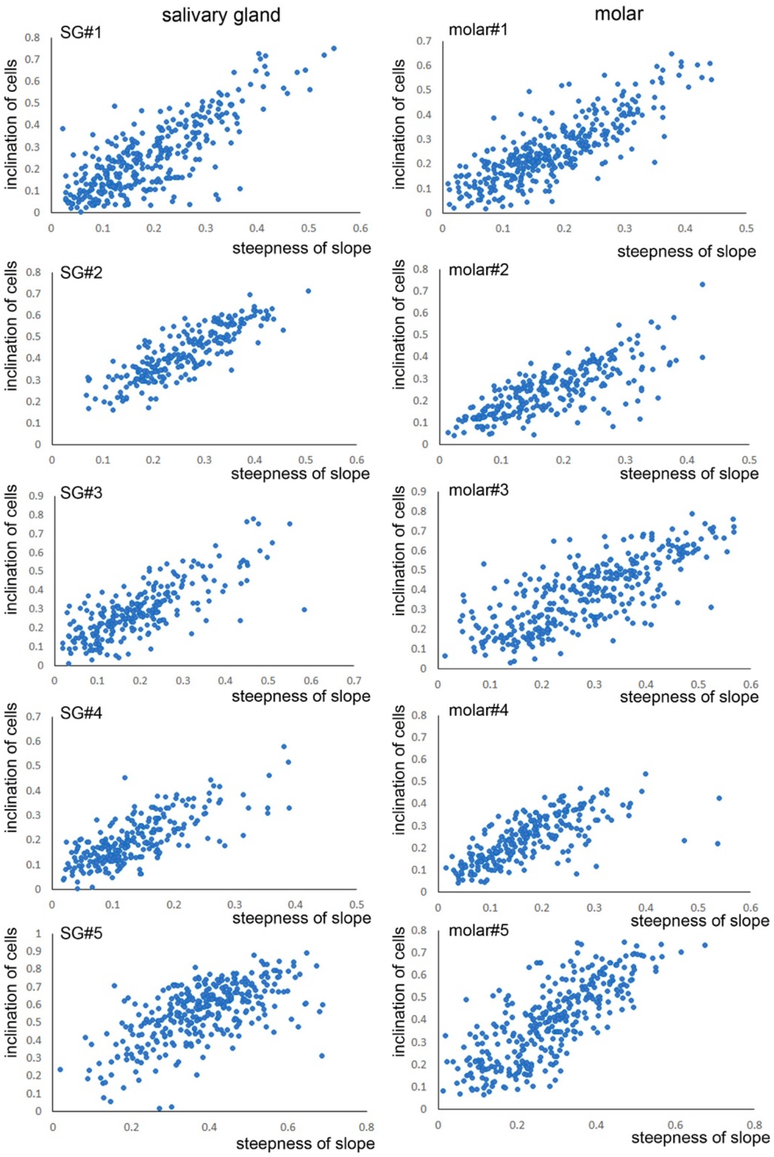

	Cell number	R-squared value
SG#1	236	0.7134
SG#2	346	0.6197
SG#3	255	0.59
SG#4	270	0.5298
SG#5	334	0.4017
Molar#1	357	0.6635
Molar#2	262	0.5672
Molar#3	333	0.579
Molar#4	246	0.5052
Molar#5	340	0.5964

Supplementary Table 1. Number of cells and Correlation coefficients for cell inclination to lamina versus lamina slope.

Supplementary Fig. 1. The inclination of epithelial cells is correlated with the steepness of the placodal slope. Amount of deviation of the cell axis from normal (perpendicular) to the basal lamina is plotted against the lamina angle-to-the-horizontal. Both are expressed as the cosine of the angle. Source data are provided as a Source Data file.

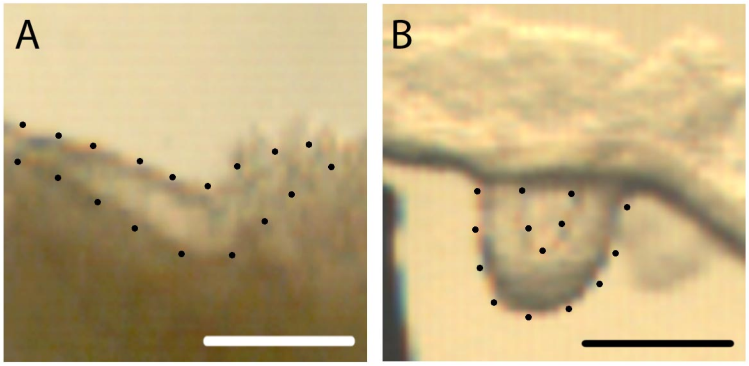

Supplementary Fig. 2. SG epithelium invagination is deeper upon enzymatic removal of underlying mesenchyme: a. Bright field image of an intact E11.5 salivary gland placode explant (frontal slice) with its underlying mesenchyme showing modest v-shaped invagination of the epithelium (apical and basal surfaces indicated by dotted lines) consistent with this stage.

b. Bright field image of an identically-staged littermate salivary gland placode enzymatically dissociated from the mesenchyme showing much deeper invagination. Scale bars = 75 µm.

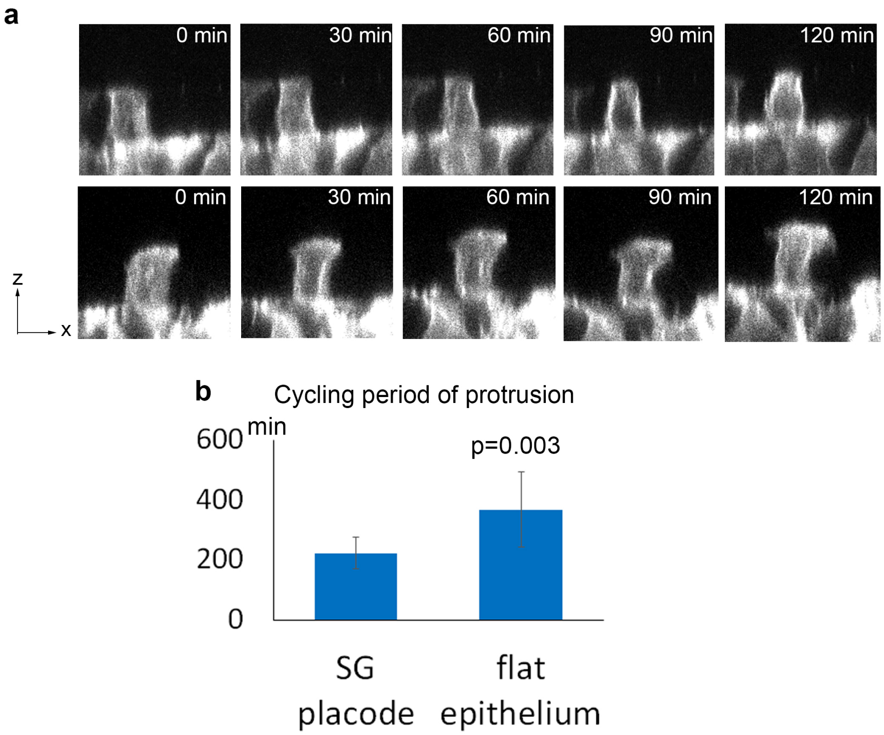

Supplementary Fig. 3. Apical planar protrusions are active in placode epithelium

a. Example stills from live imaging of a flat epithelial region. Top panels: a non-protrusive cell. Bottom panels: a protrusive cell. Scale bar = 20 µm **b**. Measurement of the persistence period of individual protrusions in cells in flat or invaginating regions. Graph shows means and SDs. Bars represent mean +/- s.d. for n = 13 and 11 cells respectively from four different placodes each. P value: two-tailed unpaired unequal variance t-test. Related to Movie S4. Source data are provided as a Source Data file.

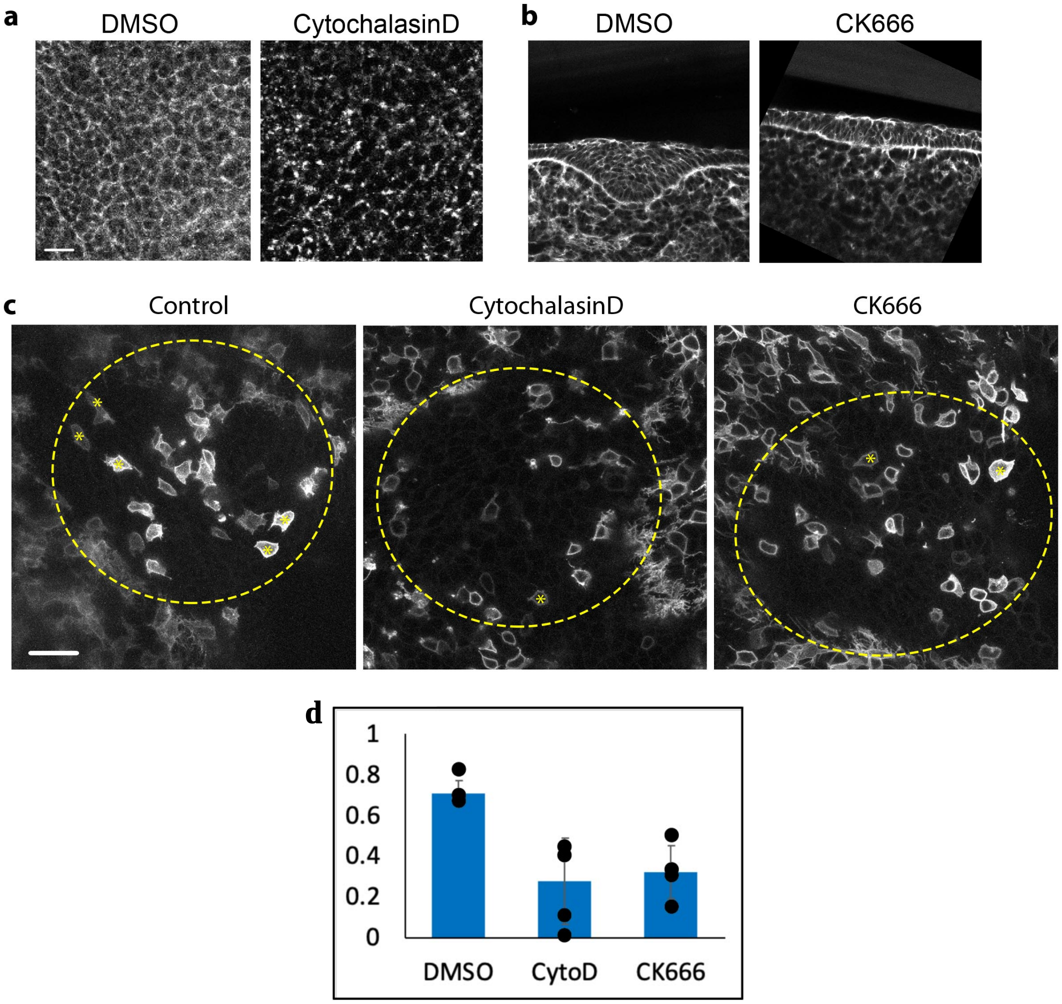

Supplementary Fig. 4. Effect of cytochalasin D and Arp2/3 inhibitor CK666 on F actin and protrusions. a. Phalloidin staining of the epithelium cultured with vehicle (DMSO), cytochalasin D (top views). **b**. Phalloidin staining of mandible slice cultured with vehicle (DMSO) and CK666. **c**. ***En face*** live images of SG placode of an mT/mG tissue treated with control vehicle, cytochalasin D or CK666. Dashed line circles: placode region. Asterisks inside cells: the cell bodies of protrusive cells. Cross: the centre of the placode. **d**. Quantification of the abundance of protrusions in the three conditions. Statistics are based on 3 independent experiments, at least 6 placodes in total in each condition. Bar graphs are means and SDs. Scale bars: 20 µm. Source data are provided as a Source Data file.
