# Supplementary material for: Epithelial invagination by a vertical telescoping cell movement in mammalian salivary glands and teeth
Source: Nat Commun. 2020 May 12;11:2366. doi: 10.1038/s41467-020-16247-z (PMC7217937; doi:10.1038/s41467-020-16247-z)
Supplement: Supplementary file 6 — Description of Additional Supplementary Files [file 41467_2020_16247_MOESM6_ESM.pdf]

Title: Supplementary Movie 1
Description: SG epithelial cells extend centripetal apical protrusions – side view Transverse view of mosaically GFP-positive cells in a SG placode, showing centripetally directed apical protrusions (arrowed). E11.25 mT/mG embryos were injected with Tamoxifen to generate mosaic labelling. Mandible explants were dissected from GFP positive embryos and mounted in DMEM/F12 medium to be imaged live en face as described in Materials and Methods. Frame interval: 30 min. 10 frames in total.

Title: Supplementary Movie 2
Description: SG epithelial cells extend centripetal apical protrusions – top view En face view of video stack in Movie S2 showing centripetal apical protrusions. Frame interval: 30 min. 10 frames in total.

Title: Supplementary Movie 3
Description: Apically protruding cell shape 3D rendering of GFP-positive basal epithelial cells on a SG slope showing characteristic oblique base and large apical protrusion.

Title: Supplementary Movie 4
Description: A few non-placodal cells have small quiescent protrusions Side view (XZ digital slice) of a mosaically labeled mandible explant in a flat region of the epithelium showing two non-placodal cells (at top) with protrusion that are essentially non-motile compared to those in the control. Frame interval: 30 min. 12 frames in total.
