# Supplementary material for: Epithelial invagination by a vertical telescoping cell movement in mammalian salivary glands and teeth
Source: Nat Commun. 2020 May 12;11:2366. doi: 10.1038/s41467-020-16247-z (PMC7217937; doi:10.1038/s41467-020-16247-z)
Supplement: Supplementary file 7 — Reporting Summary [file 41467_2020_16247_MOESM7_ESM.pdf]

nature research

Corresponding author(s): Jeremy B.A. Green

Last updated by author(s): 2020-20-02

Reporting Summary

Nature Research wishes to improve the reproducibility of the work that we publish. This form provides structure for consistency and transparency in reporting. For further information on Nature Research policies, see Authors & Referees and the Editorial Policy Checklist .

Statistics

For all statistical analyses, confirm that the following items are present in the figure legend, table legend, main text, or Methods section.

n/a	Confirmed	
☐	☒	The exact sample size (*n*) for each experimental group/condition, given as a discrete number and unit of measurement
☐	☒	A statement on whether measurements were taken from distinct samples or whether the same sample was measured repeatedly
☐	☒	The statistical test(s) used AND whether they are one- or two-sided<br>*Only common tests should be described solely by name; describe more complex techniques in the Methods section.*
☒	☐	A description of all covariates tested
☐	☒	A description of any assumptions or corrections, such as tests of normality and adjustment for multiple comparisons
☐	☒	A full description of the statistical parameters including central tendency (e.g. means) or other basic estimates (e.g. regression coefficient) AND variation (e.g. standard deviation) or associated estimates of uncertainty (e.g. confidence intervals)
☐	☒	For null hypothesis testing, the test statistic (e.g. *F*, *t*, *r*) with confidence intervals, effect sizes, degrees of freedom and *P* value noted<br>*Give P values as exact values whenever suitable.*
☒	☐	For Bayesian analysis, information on the choice of priors and Markov chain Monte Carlo settings
☒	☐	For hierarchical and complex designs, identification of the appropriate level for tests and full reporting of outcomes
☒	☐	Estimates of effect sizes (e.g. Cohen's *d*, Pearson's *r*), indicating how they were calculated

Our web collection on statistics for biologists contains articles on many of the points above.

Software and code

Policy information about availability of computer code

Data collection	Images were collected using the proprietary LAS-AF version 2.7.3 software connected to a Leica SP5 confocal microscope
Data analysis	Images were analysed using Fiji (ImageJ) version 1.52r and MATLAB version R2019a. Custom macros and code publicly accessible from the GitLab site at https://gitlab.com/jbag/cell-lamina-anglemapper (URL also given in the manuscript).

For manuscripts utilizing custom algorithms or software that are central to the research but not yet described in published literature, software must be made available to editors/reviewers. We strongly encourage code deposition in a community repository (e.g. GitHub). See the Nature Research guidelines for submitting code & software for further information.

Data

Policy information about availability of data

All manuscripts must include a data availability statement. This statement should provide the following information, where applicable:

- Accession codes, unique identifiers, or web links for publicly available datasets
- A list of figures that have associated raw data
- A description of any restrictions on data availability

The raw image, measurements and video data that support the findings of this study are available from the corresponding author upon reasonable request.

Field-specific reporting

Please select the one below that is the best fit for your research. If you are not sure, read the appropriate sections before making your selection.

Life sciences study design

All studies must disclose on these points even when the disclosure is negative.

Sample size	Sample sizes were chosen initially on logistical feasibility grounds but post-hoc tests were used to confirm that power was above the 0.80 threshold
Data exclusions	No data were excluded
Replication	Replication was achieved through both technical and biological replicates in all measurements, including sampling from independent mouse litters as well as individual embryos. Replication numbers are given for each experiment in the text and legends.
Randomization	In all experiments, embryos were chosen at random for experimentation and controls for were always contralateral matched sections/slices
Blinding	Blinding was not possible due to the small number of recognisable samples.

Reporting for specific materials, systems and methods

We require information from authors about some types of materials, experimental systems and methods used in many studies. Here, indicate whether each material, system or method listed is relevant to your study. If you are not sure if a list item applies to your research, read the appropriate section before selecting a response.

Materials & experimental systems

n/a	Involved in the study	
☐	☒	Antibodies
☒	☐	Eukaryotic cell lines
☒	☐	Palaeontology
☐	☒	Animals and other organisms
☒	☐	Human research participants
☒	☐	Clinical data

Methods

n/a	Involved in the study	
☒	☐	ChIP-seq
☒	☐	Flow cytometry
☒	☐	MRI-based neuroimaging

Antibodies

Antibodies used	All antibodies were from commercial suppliers identified in Materials & Methods together with applicable catalogue numbers. They were: anti-β-catenin 1:500 (Sigma C2206), anti-laminin 1:500 (Sigma L9393), anti-phosphorylated myosin 2 light chain (Cell Signaling 3674)<br>Lot numbers were not recorded but all targets are commonly analysed abundant proteins for which multiple sources are available.
Validation	As all the antibodies were against commonly analysed abundant proteins whose localisation in control tissues is known and was confirmed in our experiments. All had also been validated by Western blot by the manufacturer. Validation data are available on-line as follows:<br>anti-β-catenin (Sigma C2206)<br>https://www.sigmaaldrich.com/catalog/product/sigma/c2206?lang=en&region=GB<br>anti-laminin (Sigma L9393)<br>https://www.sigmaaldrich.com/catalog/product/sigma/l9393?lang=en&region=GB<br>anti-phosphorylated myosin 2 light chain (Cell Signaling 3674)<br>https://www.cellsignal.co.uk/products/primary-antibodies/phospho-myosin-light-chain-2-thr18-ser19-antibody/3674

Animals and other organisms

Policy information about studies involving animals; ARRIVE guidelines recommended for reporting animal research

Laboratory animals	"mTmG" Gt(ROSA)26Sortm4(ACTB-tdTomato,- EGFP)Luo/J (Jackson Laboratories strain 007576)<br>"tamoxifen-inducible Cre" male B6.Cg-Tg(CAG-cre/Esr1)5Amc/J (Jackson Laboratories strain 004682)<br>were used as stated in the text. For some experiments wild type CD-1 animals were used. Adult males and females were used for breeding and all other animals were embryos of ages stated in the text and of random unknown sex.
Wild animals	No wild animals were used
Field-collected samples	the study did not involve field-collected samples

Ethics oversight

As stated in the text, all animal experiments were conducted under license from the United Kingdom Home Office and approval of the authors' institutional Ethical Review Board.

Note that full information on the approval of the study protocol must also be provided in the manuscript.
